# PPARs-Orchestrated Metabolic Homeostasis in the Adipose Tissue

**DOI:** 10.3390/ijms22168974

**Published:** 2021-08-20

**Authors:** Chen Sun, Shuyu Mao, Siyu Chen, Wenxiang Zhang, Chang Liu

**Affiliations:** 1 College of Pharmacy, Xinjiang Medical University, Urumqi 830054, China; 3119030134@stu.cpu.edu.cn; 2 State Key Laboratory of Natural Medicines and School of Life Science and Technology, China Pharmaceutical University, Nanjing 211198, China; 3220030438@stu.cpu.edu.cn (S.M.); siyuchen@cpu.edu.cn (S.C.); wenxiangzhang@cpu.edu.cn (W.Z.)

**Keywords:** adipose tissue, PPAR, browning, lipid metabolism

## Abstract

It has been more than three decades since peroxisome proliferator-activated receptors (PPARs) were first discovered. Many investigations have revealed the central regulators of PPARs in lipid and glucose homeostasis in response to different nutrient conditions. PPARs have attracted much attention due to their ability to improve metabolic syndromes, and they have also been proposed as classical drug targets for the treatment of hyperlipidemia and type 2 diabetes (T2D) mellitus. In parallel, adipose tissue is known to play a unique role in the pathogenesis of insulin resistance and metabolic syndromes due to its ability to “safely” store lipids and secrete cytokines that regulate whole-body metabolism. Adipose tissue relies on a complex and subtle network of transcription factors to maintain its normal physiological function, by coordinating various molecular events, among which PPARs play distinctive and indispensable roles in adipocyte differentiation, lipid metabolism, adipokine secretion, and insulin sensitivity. In this review, we discuss the characteristics of PPARs with special emphasis on the roles of the different isotypes in adipocyte biology.

## 1. Introduction

Adipose tissue is an essential component of healthy energy homeostasis. Conversely, adipose tissue dysfunction promotes a pro-inflammatory, hyperlipidemic, and insulin-resistant environment that contributes to the pathogenesis of T2D and metabolic syndromes [1]. On the other hand, despite their obesity, some individuals appear to have a healthy metabolism. Moreover, lipodystrophy also contributes to insulin resistance and metabolic syndromes [2]. These diametrically opposite conditions illustrate the complex interplay between adipose tissue and metabolic homeostasis.

PPARs are fatty acid-activated nuclear receptors that belong to the subfamily 1 of the nuclear hormone receptor superfamily of transcription factors, and they have three subtypes: PPARα (also called NR1C1), PPARβ/δ (also called NR1C2), and PPARγ (also called NR1C3) [3]. Like other nuclear receptors, PPARs are composed of several distinct functional domains. PPARs are activated by ligands through the ligand-binding pocket in the C-terminal ligand-binding domain (LBD), which contains a ligand-dependent transactivation function (AF2), and they bind target genes through a highly conserved DNA-binding domain (DBD). In addition, the N-terminal domain (NTD, A/B domain) of PPARs contains a ligand independent activation function (AF1) that can recruit coregulatory proteins to regulate the expression of target genes. After being activated by endogenous ligands, PPARs recruit coregulator proteins with chromatin-remodeling capabilities through AF2, thereby regulating the expression of target genes [4]. The subsequent DNA binding requires dimerization with retinoid X receptor (RXR), and then the PPAR-RXR heterodimer binds to a specific DNA response element called the PPAR response element (PPRE), activating the transactivation of target genes. Meanwhile, conformational changes in PPARs, induced by diverse ligand binding, cause differential recruitment of cofactors and changes in the PPARs’ activity, thereby regulating unique physiological processes [5].

In fact, these three PPAR isoforms have some discrepancies in their functions, tissue distributions, and ligand sensitivities, in vivo. PPARα, the first rodent PPAR isoform to be identified and cloned, is expressed predominantly in the tissues that exhibit high capacity for fatty acid catabolism, such as kidneys, brown adipose tissue (BAT), liver, and skeletal muscle [6]. In these tissues, PPARα regulates the adaptive response to nutritional changes by controlling fatty acid metabolism, resulting in energy dissipation. PPARα is activated by hypolipidemic fibrates, which reduce plasma triglycerides by inhibiting the synthesis of very-low-density lipoprotein (VLDL) and increasing fatty acid oxidation in the liver [7]. PPARβ/δ was subsequently cloned from mice after the discovery of PPARα [8]. PPARβ/δ shows a relatively broader expression pattern, which is ubiquitously expressed in the heart, kidneys, skeletal muscle, fat, skin, and gastrointestinal tract, and it plays a crucial role in fatty acid and glucose metabolism [9]. PPARγ, the third member of the PPAR family, is most highly expressed in both white adipose tissue (WAT) and BAT. Due to alternative splicing and differential promoter usage, PPARγ exists as two isoforms, PPARγ1 and PPARγ2, with the former lacking the first 30 amino acids at the N-terminus, and it is expressed in a broad variety of tissues, whereas the latter is highly abundant in adipose tissue. PPARγ is mainly responsible for regulating adipocyte differentiation and lipid metabolism [10]. Thiazolidinediones (TZDs) are synthetic PPARγ ligands with robust insulin-sensitizing activities, and they are used in the treatment of type 2 diabetes [5]. Compared with the other two subtypes, PPARγ seems to play a more important role in the regulation of the biology of adipose tissue.

In this review, we highlight the roles of three PPAR isoforms in maintaining the metabolic homeostasis of adipose tissue and discuss the new findings about PPARs in adipose tissue.

## 2. Adipose Tissue Classification and Function

Adipose tissue, as a central metabolic organ, is distributed throughout the body and is composed of individual fat depots with diversity in terms of their embryology, topology, morphology, function, and gene expression profile. In mammals, WAT and BAT are the two principal types of adipose tissue. WAT is responsible for the storage and release of fat, and therefore maintains systemic energy balance and plays a role in thermal insulation, as well as in protection from mechanical damage. WAT uptakes fats and carbohydrates from the circulation and converts them into triacylglycerides (TGs) via lipogenesis. During starvation, TGs are hydrolyzed into free fatty acids (FFAs) and glycerol, which are released into the circulation to supply substrates for other tissues. On the other hand, WAT is composed of many different types of cells that secrete a variety of cytokines, chemokines, and hormones; therefore, WAT is described as an important endocrine organ in controlling the systemic energy metabolism. Adipocyte dysfunction is due to excessive lipid load causes alterations in adipokine secretion, tissue inflammation, and ectopic fat accumulation in other tissues, which subsequently cause peripheral metabolic dysfunctions, such as insulin resistance and glucose intolerance; this may explain the many adverse effects of obese states. In addition, according to its location, WAT can be roughly divided into subcutaneous WAT (sWAT) and visceral WAT (vWAT). Different lipid turnovers between sWAT and vWAT may cause distinct metabolic changes in obese states [11]. Lipid accumulation in vWAT is associated with insulin resistance and increased risk of metabolic disease, whereas lipid accumulation in sWAT may even be protective against metabolic syndromes, explaining why some people are metabolically healthy in spite of their obesity [2].

Unlike white adipocytes, which contain a large unilocular lipid droplet that fills the cytoplasm, brown adipocytes contain multilocular lipid droplets and large numbers of mitochondria for the dissipation of energy via uncoupled mitochondrial respiration. In humans, BAT can be estimated in the cervical, axillary, and paraspinal regions by using PET/CT with 2-deoxy-2-[18F] fluoroglucose [12]. BAT plays an active role in thermoregulation by converting chemical energy into heat. Cold-induced norepinephrine release stimulates lipolysis and β-oxidation in BAT. Thermogenesis is regulated by uncoupling protein 1 (UCP1), which is localized on the inner membrane of mitochondria and uncouples mitochondrial respiration from ATP synthesis. In brief, BAT is a metabolically active tissue that can clear circulating glucose and lipids; therefore, increased BAT activity is associated with several metabolic benefits, such as increased weight loss and improved glucose metabolism and insulin sensitivity.

In rodents, prolonged cold exposure leads not only to the recruitment of brown fat, but also to the appearance of white adipocytes with multilocular fat droplets and UCP1 expression, which is called “browning” [13]. These brown-like adipocytes are termed beige/brite adipocytes—the third classification of adipose tissue—and appear within classical WAT. Although beige/brite adipocytes share characteristics of brown adipocytes and express most brown-adipocyte-specific genes, such as UCP1, cell-death-inducing DNA fragmentation factor alpha subunit-like effector A (Cidea), and peroxisome proliferative activated receptor gamma coactivator 1 alpha (PGC1α), beige/brite adipocytes appear to develop from distinct populations of embryonic precursors and have distinct gene expression signatures [14].

## 3. PPARγ

**Figure 1:**
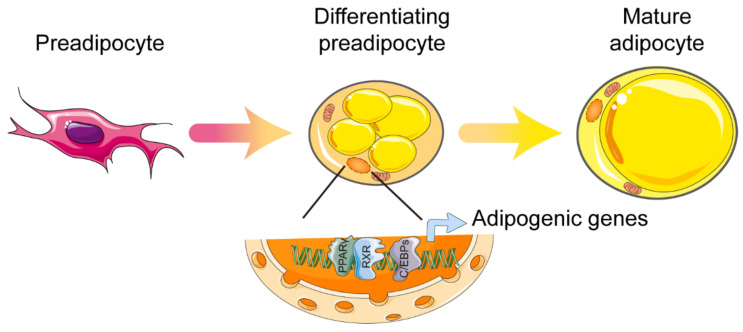
Control of white adipocyte differentiation by PPARγ. Cooperation between PPARγ and C/EBP is essential in order to fully activate the programming of mature adipocytes. Abbreviations: PPARγ, peroxisome proliferator-activated receptor γ; RXR, retinoid X receptor; C/EBPs, CCAAT/enhancer binding proteins. Figure was created using SMART–Servier Medical Art (https://smart.servier.com, the last accessed date is 29 July 2021).

PPARγ was first described as a factor induced during adipocyte differentiation, and was subsequently identified as a master regulator of adipocyte differentiation as early as 1994. These early studies indicate that PPARγ is induced and involved in adipogenesis [15,16]. In vivo studies showed that, due to placental defects, embryonic death was caused in whole-body PPARγ knockout mice. In addition, the mice that were chimeric for wild-type and PPARγ-null cells showed little or no contribution from null cells to the development of adipose tissue [17,18]. Tissue-specific gene knockout, mediated by the Cre/loxP strategy, permitted further investigation. Both adipocyte protein 2 promoter-driven Cre (aP2-Cre) and adiponectin-driven Cre (Adipoq–Cre) mouse lines were used to probe into the adipose-specific functions of PPARγ [19,20]. In these models of knockout mice, adipose tissue-specific loss of PPARγ led to critical atrophy of adipose tissue and was accompanied by significant impairment of adipokine secretion. Mechanically, the activation of the transcription factor CCAAT/enhancer binding protein (C/EBP) is one of the most important downstream effects of PPARγ during adipocyte differentiation [21]. Adipogenic transcriptional cooperation between PPARγ and C/EBP is essential in order to fully activate the programming of mature adipocytes. More than 90% of the DNA binding sites of PPARγ are also bound by C/EBP, and PPARγ relies on the induction of proteins of the C/EBP family for the complete activation of the gene transcription that is expressed in mature adipocytes (Figure 1) [22,23]. Moreover, the contributions of two PPARγ isoforms—PPARγ1 and PPARγ2—in adipogenesis are obviously different in vitro. Because the regulatory function of PPARγ2 in adipogenesis cannot be achieved by PPARγ1 in the absence of an exogenous ligand, PPARγ2 is considered the more adipogenic isoform of PPARγ [24]. In fact, the PPARγ1 isoform is sufficient for supporting development of adipose tissue and the fat deposition requirements of a lean mouse model, but the expandability of adipose tissue mainly relies on the PPARγ2 isoform under energy-excess conditions [25]. Once sufficient adipocytes are formed, mature adipocytes—along with infiltrated immune cells—secrete IL-6 and other cytokines, which, by inducing AT-rich interactive domain 5A (Arid5a), further limit the differentiation of adipocytes. Arid5a binds to the PPARγ2 promoter and prevents the activation of PPARγ2. Collectively, the feedback regulation of Arid5a and PPARγ2 maintains the homeostasis of adipose tissue. To effective adipogenesis, inhibition of Arid5a is accomplished by PPARγ2. In contrast, to limit excess adipogenesis, a check of PPARγ2 is accomplished by Arid5a [26]. In addition to its role in adipose tissue development and total storage capacity, PPARγ2 has also been identified as a crucial regulator of the lipid storage rate in adipose tissue. Mice that lack PPARγ2 cope when fat storage demands are low, but acute overfeeding overwhelms the adipose tissue, and lipids are redirected to the muscle, causing insulin resistance [27]. As already mentioned, PPARγ is essential for adipogenesis, and other adipogenic factors must act (at least in part) by activating the expression or activity of PPARγ (no transcriptional regulator that promotes adipocyte differentiation in the absence of PPARγ has been discovered).

The discovery of PPARγ mutants in human subjects also supports the important role of PPARγ in adipose tissue development [28]. In general, most of the above subjects with PPARγ mutations suffered from partial lipodystrophy, insulin resistance, and dyslipidemia. Significantly, the subcutaneous fat of limbs and the gluteal region was preferentially lost, while the visceral abdominal fat tissue was relatively preserved. In turn, treatment with PPARγ agonists in humans also resulted in redistribution of WAT [29]. In summary, PPARγ also plays a role in determining WAT distribution.

In addition to its critical role in adipogenesis, PPARγ is also indispensable for the state of mature adipocytes. Using the tamoxifen-dependent Cre-ERT2 recombination system, PPARγ was selectively ablated in adipocytes of adult mice, which resulted in the death of PPARγ-ablated adipocytes and formation of newly PPARγ-positive differentiated adipocytes within a few days [30]. Due to the compensatory effect, the remaining adipocytes were hypertrophic and more susceptible to apoptosis, which further gave rise to the presence of inflammation (such as macrophage infiltration and fibrosis) in the adipose tissue [31]. In white adipocytes, PPARγ plays a role in energy storage and adiposity. Both supraphysiological activation of PPARγ by thiazolidinediones (TZDs) and heterozygous PPARγ deficiency prevent adipocyte hypertrophy, but via different mechanisms. TZDs induce adipocyte differentiation and apoptosis, thereby increasing the number of small adipocytes, whereas the reduction of PPARγ decreases lipogenesis and promotes leptin expression in WAT [32]. On the other hand, the loss of the differentiated cell state caused by cell plasticity can result in the inability of the tissue to perform its functions. PPARγ blocks TGF-β signal transduction, thereby inhibiting the loss of adipocyte status [33].

White adipose tissue, as an important energy storage organ, strongly responds changes in nutritional signals and dynamically regulates fat storage; unsurprisingly, PPARγ also contributes to this physiological process. In adipose tissue, PPARγ expression is downregulated by fasting and insulin-deficient diabetes but induced by exposure to a high-fat diet and insulin [34,35]. The activation of PPARγ in adipocytes promotes the expression of the genes involved in the release of FFA from lipoproteins, FFA uptake, intracellular FFA transport, FFA activation, and FFA esterification [36]. Specifically, adipocytes’ lipid uptake and transport are partially regulated by lipoprotein lipase (LPL), differentiation cluster 36 (CD36), and adipocyte protein 2 (Ap2), all of which are upregulated by the response of PPARγ to TZDs treatment [37]. With the uptake of FFAs by adipocytes, PPARγ upregulates phosphoenolpyruvate carboxykinase (PEPCK), which provides a skeleton for the esterification of FFA, promotes the formation of intracellular lipid vesicles, and protects against FFA-induced lipotoxicity [38]. Furthermore, PPARγ promotes efficient storage of triglycerides in unilocular lipid droplets by regulating several lipid-droplet-associated proteins [39]. The results of the ChIP-seq experiments on the differentiated 3T3-L1 adipocytes showed that PPARγ-binding sites were found on the promoters of *Plin1*, *Plin2*, *Plin4*, *Plin5*, *Abhd5*, *Pnpla2*, *G0s2*, *Cidea*, and *Cidec* [40]. Under conditions of nutritional deficiency, PPARγ, as a fatty acid sensor, also activates lipolysis and releases FFA in order to provide maintain the balance of energy metabolism. It has been reported that the activation of PPARγ with rosiglitazone stimulates lipolysis and increases expression of adipose triglyceride lipase (ATGL) and monoacylglycerol lipase (MGL) in rat subcutaneous and visceral WAT [41]. Adipose tissue lipolysis is also stimulated by natriuretic peptides (NPs), which play a key role in maintaining blood pressure and fluid volume. Under overnutrition conditions, PPARγ upregulates high-fat diet (HFD)-dependent NP receptor C (Nprc) expression in adipocytes through long-range distal transcriptional regulation, and thereby attenuates adipocyte NP signaling in obesity [42]. Mitochondrial activity plays an important role in the health and function of adipose tissue. PPARγ induces E3 ubiquitin ligase membrane-associated RING-CH-type finger 5 (March5), which is known as an outer mitochondrial membrane protein, to regulate mitochondrial morphology and dynamics in adipocytes by controlling mitochondrial fusion. The inhibition of PPARγ expression in hypertrophic adipocytes has been observed during obesity, which may explain the decrease in mitochondrial gene expression, including that of March5 [43].

In addition to regulating lipid metabolism in WAT, PPARγ also influences the production of various signal molecules (adipokines) in white adipocytes, including adiponectin, FGF21,TNF-α, MCP-1, and resistin [44]. Adiponectin, an important adipokine, plays a cardinal role in improving obesity and metabolic diseases, and it is induced during adipocyte differentiation. PPARγ is the main regulator of adiponectin expression and processing [45]. Recent studies showed that PPARγ promotes the transport of vesicles containing adiponectin by activating reptin, which has both ATPase and DNA helicase activities. Then, upregulated transport accelerates polymerization and secretion of adiponectin, which facilitate pre-adipocyte differentiation [46]. Leptin is an adipocyte hormone that controls the mass and function of adipose tissue. By using the assay for transposase-accessible chromatin with high throughput (ATAC-seq), the functional requirement of the PPARγ-RXRα complex for the quantitative transcriptional regulation of leptin by binding to leptin regulatory element 1 (LepRE1) was confirmed. This underappreciated role of the PPARγ-RXRα complex is responsible for the quantitative control of leptin expression but does not affect its fat-specific expression [47].

Although PPARγ has been widely studied in the differentiation of WAT, it is also indispensable for the development and function of BAT. Compared with WAT, PPARγ has higher expression in both adult and embryonic BAT [48]. It was observed that PPARγ expression was already high in undifferentiated brown pre-adipocytes in vitro, and it increased further during differentiation [49,50]. Furthermore, PPARγ agonists drive BAT formation, both in vivo and in vitro [51,52]. Certainly, PPARγ is a mediator in the process of recruitment of BAT, whether by itself or in combination with other factors [53]. However, unlike in the case of WAT, C/EBPα is not a necessary factor for the gene expression of PPARγ during brown adipocyte differentiation [54]. For brown adipocytes to acquire their identity and thermogenic capacity, PPARγ recruits PR (PRD1-BF1-RIZ1 homologous) domain containing 16 (PRDM16), histone-lysine N-methyltransferase (EHMT1), and early B-cell factor (EBF2) to form a transcription complex that coordinates the transcriptional circuits toward the brown lineage. PPARγ and PRDM16 form the core part of the transcription complex, and the other two factors, EHMT1 and EBF2, are incorporated into the PPARγ-PRDM16 complex and advance its function in brown adipocytes. In detail, EHMT1, a unique methyltransferase that is specifically purified with PRDM16 by using a mass spectrum, induces the inhibitory H3K9me2 and H3K9me3 at promoter regions of the PRDM16-resident gene, which promotes precursors toward mature brown adipocytes [55]. In the same light, PPARγ recruits EBF2 to its brown-selective binding site and activates the expression of related genes, such as UCP1 [56].

Following the formation of BAT, the PPARγ-PRDM16 complex recruits a different set of cofactors in order to maintain the function of brown fat in adaptive thermogenesis and energy balance, among which PGC1α plays a central role. In brown adipocytes, PGC1α at least partially coactivates PPARγ to promote the expression of genes related to mitogenesis and thermogenesis, including *Cidea*, *Elovl3* and *Ucp1* [57]. Indeed, the PPARγ-PRDM16-PGC1α thermogenic transcription complex fine-tunes the thermogenesis and energy homeostasis by recruiting other cofactors, or it undergoes multiple modifications.

**Figure 2:**
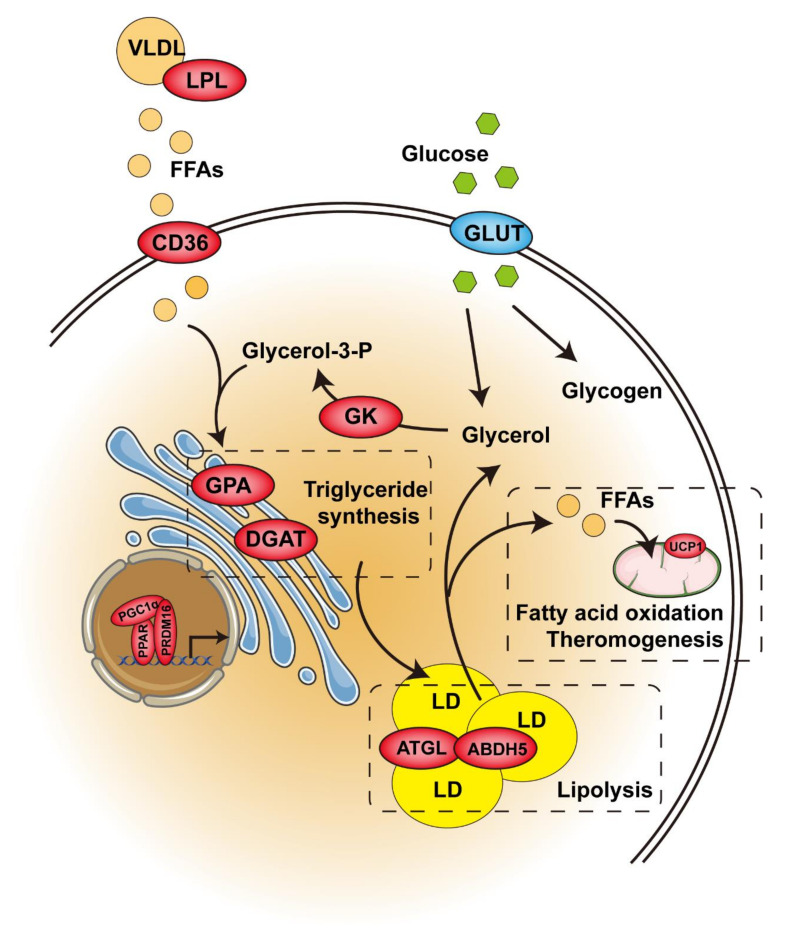
Control of glucose and lipid metabolism by PPAR in brown or beige/brite adipose tissue. Enzymes in red are activated by PPAR. The enzyme in blue remains unchanged. Abbreviations: VLDL, very-low-density lipoprotein; LPL, lipoprotein lipase; FFAs, free fatty acids; CD36, differentiation cluster 36; GPA, glycerol-3-phosphate acyltransferase; DGAT, diacylglycerol acyltransferase; GLUT, glucose transporters; GK, glycerokinase; LD, lipid droplet; ATGL, adipose triglyceride lipase; ABDH5, abhydrolase domain containing 5; UCP1, uncoupling protein 1. Figure was created using SMART–Servier Medical Art (https://smart.servier.com, the last accessed date is 29 July 2021).

The thermogenic capacity of brown adipose tissue is directly related to intracellular triglyceride storage. The hydrolysis of triglyceride provides the FFA needed for allosteric activation of UCP1, as well as for mitochondrial oxidation, which releases energy in the form of heat during thermogenesis. Interestingly, triglyceride synthesis in BAT is also significantly increased upon cold exposure [58]. Like cold exposure, pharmacological PPARγ activation significantly accelerates triglyceride synthesis, promotes hypertrophy in brown adipocytes (Figure 2), and increases BAT mass. This process is associated with upregulated absorption of fatty acids from circulating triacylglycerol via lipoprotein lipase (LPL), increased generation of glycerol 3-phosphate via glyceroneogenesis and glycerokinase (GK), and elevated esterification of fatty acids via glycerol-3-phosphate acyltransferase (GPA) and diacylglycerol acyltransferase (DGAT), which catalyze the first and last acylation of glycerol-3-phosphate, respectively [59,60,61]. On the other hand, pharmacological PPARγ activation also upregulates lipolytic genes, such as ATGL and its partner, abhydrolase domain containing 5 (Abdh5), and MGL [62]. However, the release of lipolysis-derived FFA is counteracted by its intracellular recycling and re-esterification back to TAG (Figure 2). Therefore, these higher lipase levels are not translated into higher functional lipolytic rates due to the impairment of sympathetic activity and thyroid status in this condition [63]. In addition to fatty acids, glucose is another important metabolic substrate in supporting BAT thermogenesis, which is explained by the large amount of glucose stored in brown adipocytes in the form of glycogen and the significant increase in glucose uptake caused by sympathetic nerve-mediated thermogenic activation [64]. Unlike cold exposure, pharmacological PPARγ activation dramatically reduces glucose uptake and glycogen contents, which is explained by the impairment of sympathetic activity [65]. Overall, pharmacological PPARγ activation seems to hamper the thermogenetic ability of BAT through other systemic alterations. Another option is that PPARγ is needed for β-adrenergic signaling-mediated induction of brown adipocytes, and GK is, at least in part, required for mediating PPARγ function in BAT [66]. Furthermore, it was reported that pharmacological PPARγ activation enhanced the ability of normal mice to defend against cold-induced hypothermia by switching the fuel preference of BAT from carbohydrates to lipids under cold conditions [67]. Further investigation is required in order to elucidate the mechanism of this shift.

BAT contains large numbers of mitochondria and oxidases, which are used to oxidize fatty acids and glucose in order to dissipate energy. In vivo studies showed that the activation of PPARγ by rosiglitazone was not related to the number of BAT mitochondria or the expression of PGC1α [62]. In addition, rosiglitazone did not affect the expression of PGC1α in brown adipocytes that were cultured in vitro but increased the number of mitochondria and the expression of carnitine palmitoyl transferase 1 (CPT1) [52]. However, this change increases oxygen consumption only in the presence of norepinephrine. In the other words, PPARγ cannot enhance mitochondrial function in brown adipocytes independently of the activation of the sympathetic nervous system. Interestingly, the truncated form of PPARγ2 (52 kDa), but not the full-length PPARγ2, is highly enriched in brown adipocyte mitochondria, and it regulates mtDNA-encoded ETC gene expression [68]. This unexpected regulation may provide an additional level of control for mitochondrial respiration in brown adipocytes.

The brown-like cells that are recruited through cold exposure and that arise from progenitors expressing TMEM26 and CD137 on the cell surface are referred to as beige adipocytes [69]. The activation of PPARγ by synthetic agonists induces brown fat-gene transcription in white adipocytes, both in vivo and in vitro, and these brown-like cells are referred to as brite adipocytes [70]. The significant morphological differences between brite adipocytes and beige adipocytes have been observed—the former are paucilocular, while the latter are multilocular [71]. PPARγ full agonists, such as classical TZDs, induce a brown fat phenotype in subcutaneous WAT. On the other hand, PPARγ ligands with weak or partial agonism, such as MRL24, nTZDpa, Mbx-102, and BVT.13, exhibit little or no browning effects [72]. Specifically, chronic treatment of TZDs induces activation of the PGC-1α expression [73] and stimulates a powerful stabilization of the PRDM16 Protein [72]. In vitro, brown adipocyte-like cells, which have numerous mitochondria and the presence of UCP1 protein, emerge in TZDs-treated white adipocyte cultures [70]. These cells have increased expression of not only PGC1α and UCP1, but also other brown adipocyte-specific genes, such as carnitine palmitoyltransferase 1b (CPT1B), ELOVL fatty acid elongase 3 (Elovl3), and Cidea [70]. Li and colleagues further demonstrate that PPARγ-induced WAT browning is mediated by SIRT1, PRDM16, C/EBPα and PGC1α. The transcriptional program of BAT is triggered via an SIRT1–PPARγ–PRDM16 cascade, in which PPARγ is deacetylated by SIRT1 on K268 and K293 and then recruits PRDM16 to increase the expression of BAT genes such as *Ucp1* and *Cidea* [74]. Moreover, the CDK inhibitor prevents S273 phosphorylation of PPARγ and promotes the formation of brite adipocytes in WAT [71]. In human adipocytes, kruppel-like factor 11 (KLF11) is directly induced by PPARγ and appears to cooperate with PPARγ in a feed-forward manner to activate and maintain the brite-selective gene program during long-term exposure to rosiglitazone [75]. In addition to the induction of BAT genes, the browning process also involves the repression of WAT genes. The mutation of critical amino acids within helix 7 of the PPARγ LBD suppresses TZD-mediated inhibition of WAT genes, including resistin and angiotensinogen [76]. On the molecular level, the repression of the WAT genes involves recruitment of two members of the carboxy-terminal binding protein family, CtBP1 and CtBP2, which, directed by C/EBPα, to the minimal promoter of the corresponding genes in response to the treatment of TZDs [76]. Therefore, PPARγ depends on its post-translational modifications and cofactor recruitment profiles in order to modulate its ability that activating the distinct genes subsets. However, TZD-induced browning is not associated with increased energy expenditure or weight loss in vivo, although UCP1-mediated uncoupled respiration is enhanced in adipocytes. Hence, it is important to uncouple TZDs’ benefits from their adverse effects. Further research showed that the constitutively deacetylated PPARγ (K268R/K293R) mutant mice resisted HFD-induced obesity by increasing brown remodeling in WAT, and they maintained the insulin-sensitizing response to TZD while displaying few adverse effects on fat deposition [77]. Thus, PPARγ deacetylation may dissociate the metabolic benefits of the PPARγ agonist from its adverse effects. Interestingly, the ablation of PPARγ in the sWAT of 12-month-old mice revealed PPARγ preferential regulation of brown fat gene expression for the maintenance of browning programs during aging [78].

## 4. PPARα

Unlike PPARγ, PPARα is mainly expressed in the liver, and the expression level of PPARα is low in both human and rodent WAT [79]. However, expression of PPARα in human subcutaneous and omental adipose tissue has been reported to be negatively correlated with body mass index (BMI) [80]. PPARα expression is also decreased in the WAT of mice with genetically or HFD-induced obesity, and PPARα agonists can reduce adiposity and improve insulin resistance in such obese mouse models by stimulating both differentiation and fatty acid oxidation in adipocytes [81]. Similarly, the activation of PPARα by GW7647 also stimulates differentiation and fatty acid oxidation in human adipocytes [82]. In terms of adipogenesis, the effect of PPARα seems to be partially shared with PPARγ. On the other hand, PPARα may promote a futile cycle of lipolysis and fatty acid re-esterification through the induction of GK in human white adipocytes [83]. Other studies further clarified the capacity of PPARα to promote lipolysis through several mechanisms in white adipocytes. Firstly, PPARα activation increases the expression of ATGL and HSL, which catalyze the first two important steps of lipolysis [84]. Secondly, PPARα agonists increase Ap2a2 expression, which facilitates the efficient endocytosis of β-adrenergic receptors (β-ARs) and thereby allows the avoidance of desensitization and internalization of β-ARs caused by prolonged exposure to agonists [85]. Moreover, the activation of PPARα by Wy14,643 upregulates the gene expression of adiponectin receptors (*Adipor1* and *Adipor2*) in the WAT of obese diabetic KKAγ mice [86]. In addition, PPARα has been shown to have a potent anti-inflammatory effect in white adipocytes, by inhibiting CD40 expression via upregulation of SIRT1 expression through the AMPK pathway in TNFα-treated 3T3-L1 cells [87]. All of these actions of PPARα in the WAT can enhance energy consumption and improve adipocyte hypertrophy, as well as obesity-induced insulin resistance. In addition, other studies showed that PPARα has a key role in regulating the crosstalk between the ER and mitochondria. In response to an adiponectin signal, PPARα binds to the activating transcription factor-2 (ATF2) promoter region, resulting in the inhibition of ATF2 transcription, thereby alleviating ER stress and apoptosis in adipocytes [88].

As PPARα is the key regulator of cellular fatty acid uptake and oxidation (Figure 2), it is not surprising that PPARα is highly expressed in BAT. PPARα-deficient mice, despite having a normal BAT morphology, exhibited a thermogenesis-associated disorder in response to cold exposure. [89] Furthermore, compared with PPARα-deficient mice, liver-specific PPARα-null mice had more severe hypothermia after 24h-fasting, indicating that extrahepatic PPARα is necessary for maintaining whole-body temperature [90]. The possible explanation includes, but is not limited to, the role of PPARα in BAT. PPARα is activated by endogenous ligands that are derived from cold-induced lipolysis, and it upregulates the fatty acid oxidation and thermogenic genes through a cooperative mechanism with PGC1α. This regulation is initiated by the positive feedback loop of PPARα-induced PGC1α expression. In addition, PPARα regulates the expression of PRDM16, which binds to the PPARα-binding site of the PGC1α gene promoter and enhances PGC1α expression [91]. The energy source used for ATP production or thermogenesis is mainly supplied by glucose or fatty acids in BAT. Pyruvate dehydrogenases (PDH) and pyruvate dehydrogenase kinases (PDK) play a key role in the process of supplying energy from glucose. The activated PPARα competes with hepatocyte nuclear factor 4 alpha (HNF4α), a positive regulator, to bind the PDHβ promoter region, thereby suppressing PDHβ expression during cold exposure [92]. This finding agrees with the above-mentioned switching of fuel preference in BAT under cold conditions. Moreover, PPARα activation resulted in a reversal of whitening, with the favored thermogenesis being sustained by enhanced β3-adrenergic stimulation, lipolysis, and β-oxidation in BAT of the mice with HFD-induced obesity [93]. However, recent studies have suggested that PPARα is dispensable in thermogenesis. Even though PPARα agonists enhance the function of BAT, PPARα depletion in BAT did not affect the expression of classic BAT markers (such as *Ucp1*, *Cidea* and *Cox7a1*) [66]. In addition, WT and PPARα-null mice had no differences in BAT function during CL316,243 (β3-adrenergic agonist) treatment [94]. Furthermore, another study confirmed that the redundancy of PPARα with PPARγ is because PPARα binds to a subset of PPARγ sites [95].

Indeed, in vitro PPARα activation in human white adipocytes induces the expression of brown adipocyte-selective genes, such as *PGC1α*, *PRDM16, UCP1* and *DIO2* [91]. In addition, the activation of PPARα by chronic fenofibrate administration increases the gene expression of PGC1α and irisin, and it yields UCP-1-positive beige cells in the sWAT of the mice with HFD-induced obesity [96]. Furthermore, PPARα-null mice displayed a disrupted induction of thermogenic and brite markers in the inguinal WAT upon β3-adrenergic agonist treatment, which was associated with lower PDK4 expression [94]. Taken together, these data indicate that PPARα activation can promote the browning of WAT. Surprisingly, recent research showed that PPARα, despite its marked upregulation by cold in inguinal WAT, is completely dispensable for cold-induced browning in mice because cold-induced changes in gene expression in inguinal WAT are fully maintained in the absence of PPARα [97]. The reason why PPARα is induced by cold exposure, if it is not involved in regulating gene expression in inguinal WAT, remains unknown. The possible explanation is that PPARα activation is a sufficient and unnecessary condition during WAT browning.

## 5. PPARβ/δ

Similar to PPARγ, the expression of PPARβ/δ is also upregulated during adipocyte differentiation, and the difference is that PPARγ is expressed at the late stage of differentiation whereas PPARβ/δ is expressed in the initial stage of differentiation [98]. In vitro, PPARβ/δ promotes preadipocyte differentiation by inducing the expression of adipogenesis-related genes, such as PPARγ and fatty acid transporter (FAT) [99]. Another study showed that PPARβ/δ plays an important role in the proliferation of adipocyte precursor cells, and it has only a minor impact on terminal adipocyte differentiation [100]. This finding is consistent with the observation that PPARβ/δ knockout mice had impaired gonadal adipose stores [101]. On the other hand, the activation of PPARβ/δ promotes fatty acid oxidation and energy uncoupling in vivo and in vitro [102]. Moreover, PPARβ/δ prevents angiotensin-II-induced adipocyte hypertrophy and stimulates adipocyte remodeling with smaller adipocytes, while decreasing inflammation and increasing adiponectin secretion via the activation of haem oxygenase-1 (HO-1) expression and the Wnt-canonical pathway [103]. The activation of PPARβ/δ by GW501516 arrested IL-6–dependent reduction in insulin-stimulated Akt phosphorylation and glucose uptake by inhibiting ERK1/2 and inhibiting the activation of the signal transducer and activator of transcription-3 (STAT3) and the upregulation of the suppressor of cytokine signaling 3 (SOCS3) [104]. Otherwise, in adipose tissue-resident macrophages, PPARβ/δ is upregulated by IL-13 to control the polarization of macrophages toward alternative activation, thereby improving insulin sensitivity [105]. Furthermore, PPARβ/δ ablation impairs macrophage M2 polarization, which, in turn, causes inflammation and results in the stimulation of lipolysis and insulin resistance in adipocytes.

In BAT, PPARβ/δ activation induces the expression of genes related to fatty acid oxidation and thermogenesis (Figure 2) [102]. Furthermore, BAT-specific PPARβ/δ knockout mice are compromised with respect to maintaining body temperature during cold exposure, because PGC1α no longer binds to the UCP1 promoter in the absence of PPARβ/δ. Interestingly, PPARβ/δ not only mediates the actions of PGC1α, but also regulates the expression of twist family BHLH transcription factor 1 (twist-1), which inhibits histone H3 acetylation on the promoters of PGC1α target genes, suggesting a negative-feedback regulatory mechanism [106].

As a nutritional signal sensor, PPARβ/δ has been described as a candidate for the induction of adiposal browning [102]. However, in mice with HFD-induced obesity, pharmacological PPARβ/δ activation tackles glucose intolerance and reduces adipocyte size, but not positive UCP1 beige adipocytes were not shown in the sWAT, which may have been because of the enhanced Cidea gene expression, which inhibited the activity of UCP1 by forming a complex [107]. On the other hand, a recent study showed that PPARβ/δ mediates leptin-induced FGF21 expression in the crosstalk of brain–visceral adipose tissue, therefore contributing to the white-to-beige cell transition in WAT via autocrine/paracrine mechanisms [108].

## 6. Conclusions

**Figure 3:**
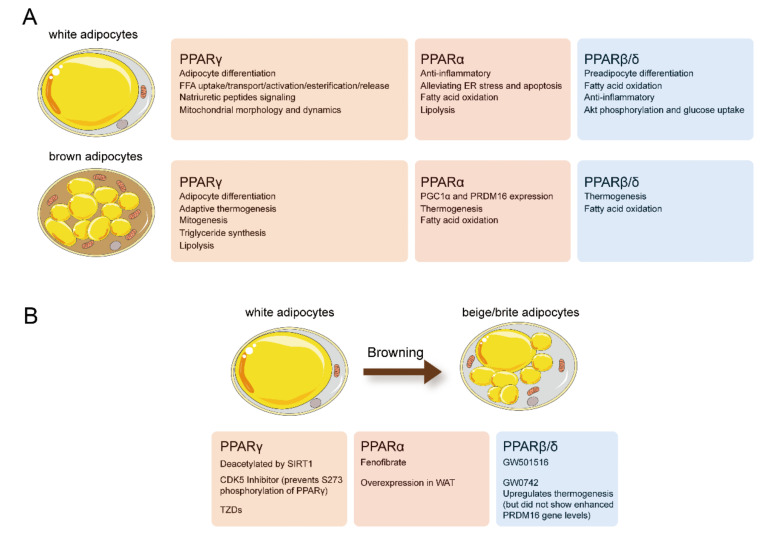
(**A**) Effects of physiologic and pharmacologic PPAR activation on white and brown adipocyte biology. (**B**) Physiologic and pharmacologic PPAR activation promote the “browning” of WAT. Figure was created using SMART–Servier Medical Art (https://smart.servier.com, the last accessed date is 29 July 2021).

Numerous studies support the crucial role of PPARs in maintaining metabolic homeostasis in adipose tissue (Figure 3). PPARγ plays a key role in adipocyte differentiation, and lipid storage, PPARα and PPARβ/δ are primarily involved in adaptive thermogenesis and lipid utilization in adipose tissue. Selective and potent PPARα or PPARγ agonists enhance the activity of BAT and induce the “browning “of WAT (Figure 2). However, over the last decades, market withdrawal and the failure of drug development programs have made people doubt the clinical value of compounds with PPAR-activation functions. Meanwhile, the PPARγ agonist rosiglitazone and dual PPAR agonists displayed ineluctable adverse effects that led to restricted use or halted development. Nevertheless, most of these side effects were either caused by nonspecific and off-target effects of the drugs or excessive PPARγ activation. With the development of targeted therapy, PPAR targeted therapy will regain its brilliance.

At present, significant advances have been made in understanding the sophistication of the function of adipose tissue and the role of adipose tissue in controlling systemic energy balance. Obviously, targeting adipose tissue is an effective strategy for the treatment of obesity, insulin resistance, T2D, and another metabolic syndromes. In adipose tissue, PPARs represent how various metabolic signaling networks converge into a single nuclear factor, and the transcriptional activity of PPARs is controlled by multiple regulative layers of alternative splicing, post-translational modification, and coactivator/suppressor interaction, thereby resulting in its time- and tissue-specific responses. The study of alternative splicing and post-translational modification of PPARs in adipose tissue under diverse physiological and pathological conditions still needs further research. Altogether, we are convinced that the targeting of adipose PPARs in metabolic disorders remains a valuable and promising approach, with a future ahead of it.

## Data Availability

Not applicable.

## References

[1] Chait A., de Hartigh L.J. (2020). Adipose Tissue Distribution, Inflammation and Its Metabolic Consequences, Including Diabetes and Cardiovascular Disease. Front. Cardiovasc. Med..

[2] Kahn C.R., Wang G., Lee K.Y. (2019). Altered adipose tissue and adipocyte function in the pathogenesis of metabolic syndrome. J. Clin. Investig..

[3] Brunmeir R., Xu F. (2018). Functional Regulation of PPARs through Post-Translational Modifications. Int. J. Mol. Sci..

[4] Cataldi S., Costa V., Ciccodicola A., Aprile M. (2021). PPARγ and Diabetes: Beyond the Genome and Towards Personalized Medicine. Curr. Diabetes Rep..

[5] Ahmadian M., Suh J.M., Hah N., Liddle C., Atkins A.R., Downes M., Evans R.M. (2013). PPARγ signaling and metabolism: The good, the bad and the future. Nat. Med..

[6] Issemann I., Green S. (1990). Activation of a member of the steroid hormone receptor superfamily by peroxisome proliferators. Nature.

[7] Okopień B., Bułdak Ł., Bołdys A. (2018). Benefits and risks of the treatment with fibrates—A comprehensive summary. Expert Rev. Clin. Pharmacol..

[8] Kliewer S.A., Forman B.M., Blumberg B., Ong E.S., Borgmeyer U., Mangelsdorf D., Umesono K., Evans R. (1994). Differential expression and activation of a family of murine peroxisome proliferator-activated receptors. Proc. Natl. Acad. Sci. USA.

[9] Bedu E., Wahli W., Desvergne B. (2005). Peroxisome proliferator-activated receptor β/δ as a therapeutic target for metabolic diseases. Expert Opin. Ther. Targets.

[10] Corrales P., Vidal-Puig A., Medina-Gómez G. (2018). PPARs and Metabolic Disorders Associated with Challenged Adipose Tissue Plasticity. Int. J. Mol. Sci..

[11] Spalding K.L., Bernard S., Näslund E., Salehpour M., Possnert G., Appelsved L., Fu K.-Y., Alkass K., Druid H., Thorell A. (2017). Impact of fat mass and distribution on lipid turnover in human adipose tissue. Nat. Commun..

[12] Bhatt P.S., Dhillo W.S., Salem V. (2017). Human brown adipose tissue—Function and therapeutic potential in metabolic disease. Curr. Opin. Pharmacol..

[13] Bartelt A., Heeren J. (2014). Adipose tissue browning and metabolic health. Nat. Rev. Endocrinol..

[14] Schoettl T., Fischer I.P., Ussar S. (2018). Heterogeneity of adipose tissue in development and metabolic function. J. Exp. Biol..

[15] Chawla A., Schwarz E.J., Dimaculangan D.D., Lazar M.A. (1994). Peroxisome proliferator-activated receptor (PPAR) gamma: Adipose-predominant expression and induction early in adipocyte differentiation. Endocrinology.

[16] Tontonoz P., Hu E., Graves R.A., Budavari A.I., Spiegelman B.M. (1994). mPPAR gamma 2: Tissue-specific regulator of an adipocyte enhancer. Genes Dev..

[17] Rosen E.D., Sarraf P., Troy A.E., Bradwin G., Moore K., Milstone D.S., Spiegelman B.M., Mortensen R.M. (1999). PPAR gamma is required for the differentiation of adipose tissue in vivo and in vitro. Mol. Cell.

[18] Barak Y., Nelson M.C., Ong E.S., Jones Y.Z., Ruiz-Lozano P., Chien K.R., Koder A., Evans R.M. (1999). PPAR gamma is required for placental, cardiac, and adipose tissue development. Mol. Cell.

[19] Jones J.R., Barrick C., Kim K.-A., Lindner J., Blondeau B., Fujimoto Y., Shiota M., Kesterson R.A., Kahn B.B., Magnuson M.A. (2005). Deletion of PPARgamma in adipose tissues of mice protects against high fat diet-induced obesity and insulin resistance. Proc. Natl. Acad. Sci. USA.

[20] Wang F., Mullican S.E., Dispirito J.R., Peed L.C., Lazar M.A. (2013). Lipoatrophy and severe metabolic disturbance in mice with fat-specific deletion of PPARγ. Proc. Natl. Acad. Sci. USA.

[21] Ghaben A.L., Scherer P.E. (2019). Adipogenesis and metabolic health. Nat. Rev. Mol. Cell Biol..

[22] Lee J.-E., Schmidt H., Lai B., Ge K. (2019). Transcriptional and Epigenomic Regulation of Adipogenesis. Mol. Cell. Biol..

[23] Lefterova M.I., Zhang Y., Steger D.J., Schupp M., Schug J., Cristancho A., Feng D., Zhuo D., Stoeckert C.J., Liu X.S. (2008). PPARγ and C/EBP factors orchestrate adipocyte biology via adjacent binding on a genome-wide scale. Genes Dev..

[24] Ren D., Collingwood T.N., Rebar E.J., Wolffe A.P., Camp H.S. (2002). PPARγ knockdown by engineered transcription factors: Exogenous PPARγ2 but not PPARγ1 reactivates adipogenesis. Genes Dev..

[25] Medina-Gomez G., Gray S.L., Yetukuri L., Shimomura K., Virtue S., Campbell M., Curtis R.K., Jimenez-Linan M., Blount M., Yeo G.S.H. (2007). PPAR gamma 2 Prevents Lipotoxicity by Controlling Adipose Tissue Expandability and Peripheral Lipid Metabolism. PLoS Genet..

[26] Chalise J.P., Hashimoto S., Parajuli G., Kang S., Singh S.K., Gemechu Y., Metwally H., Nyati K.K., Dubey P.K., Zaman M.M.-U. (2019). Feedback regulation of Arid5a and Ppar-γ2 maintains adipose tissue homeostasis. Proc. Natl. Acad. Sci. USA.

[27] Virtue S., Petkevicius K., Moreno-Navarrete J.M., Jenkins B., Hart D., Dale M., Koulman A., Fernández-Real J.M., Vidal-Puig A. (2018). Peroxisome Proliferator-Activated Receptor γ2 Controls the Rate of Adipose Tissue Lipid Storage and Determines Metabolic Flexibility. Cell Rep..

[28] Mann J.P., Savage D.B. (2019). What lipodystrophies teach us about the metabolic syndrome. J. Clin. Investig..

[29] Semple R.K., Chatterjee V.K., O’Rahilly S. (2006). PPAR gamma and human metabolic disease. J. Clin. Investig..

[30] Imai T., Takakuwa R., Marchand S., Dentz E., Bornert J.-M., Messaddeq N., Wendling O., Mark M., Desvergne B., Wahli W. (2004). Peroxisome proliferator-activated receptor γ is required in mature white and brown adipocytes for their survival in the mouse. Proc. Natl. Acad. Sci. USA.

[31] He W., Barak Y., Havener A., Olson P., Liao D., Le J., Nelson M., Ong E., Olefsky J.M., Evans R.M. (2003). Adipose-specific peroxisome proliferator-activated receptor γ knockout causes insulin resistance in fat and liver but not in muscle. Proc. Natl. Acad. Sci. USA.

[32] Yamauchi T., Kamon J., Waki H., Murakami K., Motojima K., Komeda K., Ide T., Kubota N., Terauchi Y., Tobe K. (2001). The Mechanisms by Which Both Heterozygous Peroxisome Proliferator-activated Receptor γ (PPARγ) Deficiency and PPARγ Agonist Improve Insulin Resistance. J. Biol. Chem..

[33] Taylor B., Shah A., Bielczyk-Maczyńska E. (2020). TGF-β is insufficient to induce adipocyte state loss without concurrent PPARγ downregulation. Sci. Rep..

[34] Rieusset J., Andreelli F., Auboeuf D., Roques M., Vallier P., Riou J.P., Auwerx J., Laville M., Vidal H. (1999). Insulin acutely regulates the expression of the peroxisome proliferator-activated receptor-gamma in human adipocytes. Diabetes.

[35] Vidal-Puig A., Jimenez-Liñan M., Lowell B.B., Hamann A., Hu E., Spiegelman B., Flier J.S., Moller D. (1996). Regulation of PPAR gamma gene expression by nutrition and obesity in rodents. J. Clin. Investig..

[36] Tontonoz P., Spiegelman B.M. (2008). Fat and Beyond: The Diverse Biology of PPARgamma. Annu. Rev. Biochem..

[37] Skat-Rordam J., Hojland Ipsen D., Lykkesfeldt J., Tveden-Nyborg P. (2019). A role of peroxisome proliferator-activated receptor gamma in non-alcoholic fatty liver disease. Basic Clin. Pharmacol. Toxicol..

[38] Li J., Liu Y.-P. (2018). The roles of PPARs in human diseases. Nucleosides Nucleotides Nucleic Acids.

[39] Rodriguez M.A.D.L.R., Kersten S. (2017). Regulation of lipid droplet-associated proteins by peroxisome proliferator-activated receptors. Biochim. Biophys. Acta BBA—Mol. Cell Biol. Lipids.

[40] Christian M. (2013). Nuclear receptor-mediated regulation of lipid droplet-associated protein gene expression in adipose tissue. Horm. Mol. Biol. Clin. Investig..

[41] Festuccia W.T., Laplante M., Berthiaume M., Gelinas Y., Deshaies Y. (2006). PPARgamma agonism increases rat adipose tissue lipolysis, expression of glyceride lipases, and the response of lipolysis to hormonal control. Diabetologia.

[42] Shi F., Simandi Z., Nagy L., Collins S. (2021). Diet-dependent natriuretic peptide receptor C expression in adipose tissue is mediated by PPARγ via long-range distal enhancers. J. Biol. Chem..

[43] Bond S., Moody S., Liu Y., Civelek M., Villanueva C., Gregorevic P., Kingwell B.A., Hevener A.L., Lusis A.J., Henstridge D.C. (2019). The E3 ligase MARCH5 is a PPARγ target gene that regulates mitochondria and metabolism in adipocytes. Am. J. Physiol. Endocrinol. Metab..

[44] Muise E.S., Azzolina B., Kuo D.W., El-Sherbeini M., Tan Y., Yuan X., Mu J., Thompson J.R., Berger J.P., Wong K.K. (2008). Adipose Fibroblast Growth Factor 21 Is Up-Regulated by Peroxisome Proliferator-Activated Receptor γ and Altered Metabolic States. Mol. Pharmacol..

[45] Astapova O., Leff T. (2012). Adiponectin and PPARγ: Cooperative and interdependent actions of two key regulators of metabolism. Vitam. Horm..

[46] Zhu D., Xu L., Wei X., Xia B., Gong Y., Li Q., Chen X. (2020). PPARγ enhanced Adiponectin polymerization and trafficking by promoting RUVBL2 expression during adipogenic differentiation. Gene.

[47] Zhang Y., Dallner O.S., Nakadai T., Fayzikhodjaeva G., Friedman J.M. (2018). A non-canonical-PPARγ/RXRα-binding sequence regulates leptin expression in response to changes in adipose tissue mass. Proc. Natl. Acad. Sci. USA.

[48] Tymciw T. (2018). Hormonal and Temporal Regulation of Adipogenic Genes in Classical Brown Adipocytes. Master’s Thesis.

[49] Lindgren E.M., Nielsen R., Petrovic N., Jacobsson A., Mandrup S., Cannon B., Nedergaard J. (2004). Noradrenaline represses PPAR (peroxisome-proliferator-activated receptor) γ2 gene expression in brown adipocytes: Intracellular signalling and effects on PPARγ2 and PPARγ1 protein levels. Biochem. J..

[50] Valmaseda A., Carmona M., Barberá M., Viñas O., Mampel T., Iglesias R., Villarroya F., Giralt M. (1999). Opposite regulation of PPAR-α and -γ gene expression by both their ligands and retinoic acid in brown adipocytes. Mol. Cell. Endocrinol..

[51] Tai T.-A.C., Jennermann C., Brown K.K., Oliver B.B., MacGinnitie M.A., Wilkison W.O., Brown H.R., Lehmann J.M., Kliewer S.A., Morris D.C. (1996). Activation of the Nuclear Receptor Peroxisome Proliferator-activated Receptor γ Promotes Brown Adipocyte Differentiation. J. Biol. Chem..

[52] Petrovic N., Shabalina I., Timmons J.A., Cannon B., Nedergaard J. (2008). Thermogenically competent nonadrenergic recruitment in brown preadipocytes by a PPARγ agonist. Am. J. Physiol. Endocrinol. Metab..

[53] Oelkrug R., Polymeropoulos E.T., Jastroch M. (2015). Brown adipose tissue: Physiological function and evolutionary significance. J. Comp. Physiol. B Biochem. Syst. Environ. Physiol..

[54] Linhart H.G., Ishimura-Oka K., DeMayo F., Kibe T., Repka D., Poindexter B., Bick R.J., Darlington G.J. (2001). C/EBPα is required for differentiation of white, but not brown, adipose tissue. Proc. Natl. Acad. Sci. USA.

[55] Nagano G., Ohno H., Oki K., Kobuke K., Shiwa T., Yoneda M., Kohno N. (2015). Activation of Classical Brown Adipocytes in the Adult Human Perirenal Depot Is Highly Correlated with PRDM16–EHMT1 Complex Expression. PLoS ONE.

[56] Rajakumari S., Wu J., Ishibashi J., Lim H.-W., Giang A.-H., Won K.J., Reed R.R., Seale P. (2013). EBF2 Determines and Maintains Brown Adipocyte Identity. Cell Metab..

[57] Spiegelman B.M., Puigserver P., Wu Z. (2000). Regulation of adipogenesis and energy balance by PPARgamma and PGC-1. Int. J. Obes..

[58] Moura M.A., Festuccia W.T.L., Kawashita N.H., Garofalo M.A.R., Brito S.R.C., Kettelhut I.C., Migliorini R.H. (2005). Brown adipose tissue glyceroneogenesis is activated in rats exposed to cold. Pflügers Arch..

[59] Festuccia W.T., Deshaies Y. (2009). Depot specificities of PPARγ ligand actions on lipid and glucose metabolism and their implication in PPARγ-mediated body fat redistribution. Clin. Lipidol..

[60] Festuccia W.T., Blanchard P.-G., Turcotte V., Laplante M., Sariahmetoglu M., Brindley D.N., Richard D., Deshaies Y. (2009). The PPARγ agonist rosiglitazone enhances rat brown adipose tissue lipogenesis from glucose without altering glucose uptake. Am. J. Physiol. Regul. Integr. Comp. Physiol..

[61] Laplante M., Festuccia W.T., Soucy G., Blanchard P.-G., Renaud A., Berger J.P., Olivecrona G., Deshaies Y. (2009). Tissue-specific postprandial clearance is the major determinant of PPARγ-induced triglyceride lowering in the rat. Am. J. Physiol. Regul. Integr. Comp. Physiol..

[62] Festuccia W.T., Blanchard P.-G., Richard D., Deshaies Y. (2010). Basal adrenergic tone is required for maximal stimulation of rat brown adipose tissue UCP1 expression by chronic PPAR-γ activation. Am. J. Physiol. Regul. Integr. Comp. Physiol..

[63] Festuccia W.T., Öztezcan S., Laplante M., Berthiaume M., Michel C., Dohgu S., Denis R.G., Brito M.N., Brito N.A., Miller D.S. (2008). Peroxisome Proliferator-Activated Receptor-γ-Mediated Positive Energy Balance in the Rat Is Associated with Reduced Sympathetic Drive to Adipose Tissues and Thyroid Status. Endocrinology.

[64] Yau W.W., Yen P.M. (2020). Thermogenesis in Adipose Tissue Activated by Thyroid Hormone. Int. J. Mol. Sci..

[65] Festuccia W.T., Blanchard P.G., Deshaies Y. (2011). Control of Brown Adipose Tissue Glucose and Lipid Metabolism by PPARgamma. Front. Endocrinol..

[66] Lasar D., Rosenwald M., Kiehlmann E., Balaz M., Tall B., Opitz L., Lidell M.E., Zamboni N., Krznar P., Sun W. (2018). Peroxisome Proliferator Activated Receptor Gamma Controls Mature Brown Adipocyte Inducibility through Glycerol Kinase. Cell Rep..

[67] Gao R., Chen W., Yan H., Xie X., Liu D., Wu C., Zhu Z., Li H., Dong F., Wang L. (2018). PPARγ agonist rosiglitazone switches fuel preference to lipids in promoting thermogenesis under cold exposure in C57BL/6 mice. J. Proteom..

[68] Chang J.S., Ha K. (2018). A truncated PPAR gamma 2 localizes to mitochondria and regulates mitochondrial respiration in brown adipocytes. PLoS ONE.

[69] Wu J., Boström P., Sparks L.M., Ye L., Choi J.H., Giang A.-H., Khandekar M., Virtanen K.A., Nuutila P., Schaart G. (2012). Beige Adipocytes Are a Distinct Type of Thermogenic Fat Cell in Mouse and Human. Cell.

[70] Petrovic N., Walden T.B., Shabalina I., Timmons J.A., Cannon B., Nedergaard J. (2010). Chronic Peroxisome Proliferator-activated Receptor γ (PPARγ) Activation of Epididymally Derived White Adipocyte Cultures Reveals a Population of Thermogenically Competent, UCP1-containing Adipocytes Molecularly Distinct from Classic Brown Adipocytes. J. Biol. Chem..

[71] Wang H., Liu L., Lin J.Z., Aprahamian T., Farmer S.R. (2016). Browning of White Adipose Tissue with Roscovitine Induces a Distinct Population of UCP1 + Adipocytes. Cell Metab..

[72] Ohno H., Shinoda K., Spiegelman B.M., Kajimura S. (2012). PPARγ agonists Induce a White-to-Brown Fat Conversion through Stabilization of PRDM16 Protein. Cell Metab..

[73] Wilson-Fritch L., Nicoloro S., Chouinard M., Lazar M.A., Chui P.C., Leszyk J., Straubhaar J., Czech M.P., Corvera S. (2004). Mitochondrial remodeling in adipose tissue associated with obesity and treatment with rosiglitazone. J. Clin. Investig..

[74] Qiang L., Wang L., Kon N., Zhao W., Lee S., Zhang Y., Rosenbaum M., Zhao Y., Gu W., Farmer S. (2012). Brown Remodeling of White Adipose Tissue by SirT1-Dependent Deacetylation of Pparγ. Cell.

[75] Loft A., Forss I., Siersbæk M.S., Schmidt S.F., Larsen A.-S.B., Madsen J.G.S., Pisani D.F., Nielsen R., Aagaard M.M., Mathison A. (2015). Browning of human adipocytes requires KLF11 and reprogramming of PPARγ superenhancers. Genes Dev..

[76] Vernochet C., Peres S.B., Davis K.E., McDonald M.E., Qiang L., Wang H., Scherer P.E., Farmer S.R. (2009). C/EBPα and the Corepressors CtBP1 and CtBP2 Regulate Repression of Select Visceral White Adipose Genes during Induction of the Brown Phenotype in White Adipocytes by Peroxisome Proliferator-Activated Receptor γ Agonists. Mol. Cell. Biol..

[77] Kraakman M.J., Liu Q., Postigo-Fernandez J., Ji R., Kon N., Larrea D., Namwanje M., Fan L., Chan M., Area-Gomez E. (2018). PPARγ deacetylation dissociates thiazolidinedione’s metabolic benefits from its adverse effects. J. Clin. Investig..

[78] Xu L., Ma X., Verma N.K., Wang D., Gavrilova O., Proia R.L., Finkel T., Mueller E. (2018). Ablation of PPAR γ in subcutaneous fat exacerbates age-associated obesity and metabolic decline. Aging Cell.

[79] Auboeuf D., Rieusset J., Fajas L., Vallier P., Frering V., Riou J.P., Staels B., Auwerx J., Laville M., Vidal H. (1997). Tissue distribution and quantification of the expression of mRNAs of peroxisome proliferator–activated receptors and liver X receptor-α in humans: No alteration in adipose tissue of obese and NIDDM patients. Diabetes.

[80] MacLaren R., Cui W., Simard S., Cianflone K. (2008). Influence of obesity and insulin sensitivity on insulin signaling genes in human omental and subcutaneous adipose tissues. J. Lipid Res..

[81] Goto T., Lee J.-Y., Teraminami A., Kim Y.-I., Hirai S., Uemura T., Inoue H., Takahashi N., Kawada T. (2011). Activation of peroxisome proliferator-activated receptor-alpha stimulates both differentiation and fatty acid oxidation in adipocytes. J. Lipid Res..

[82] Lee J.-Y., Hashizaki H., Goto T., Sakamoto T., Takahashi N., Kawada T. (2011). Activation of peroxisome proliferator-activated receptor-α enhances fatty acid oxidation in human adipocytes. Biochem. Biophys. Res. Commun..

[83] Mazzucotelli A., Vigueri N., Tiraby C., Annicotte J.-S., Mairal A., Klimcakova E., Lepin E., Delmar P., Dejean S., Tavernier G. (2007). The transcriptional coactivator peroxisome proliferator–activated receptor (PPAR) γ coactivator-1α and the nuclear receptor PPARα control the expression of glycerol kinase and metabolism genes independently of PPARγ activation in human white adipocytes. Diabetes.

[84] Miranda J., Lasa A., Fernández-Quintela A., García-Marzo C., Ayo J., Dentin R., Portillo M.P. (2011). *cis*-9, *trans*-11, *cis*-15 and *cis*-9, *trans*-13, *cis*-15 CLNA Mixture Activates PPARα in HEK293 and Reduces Triacylglycerols in 3T3-L1 cells. Lipids.

[85] Montgomery M.K., Bayliss J., Keenan S., Rhost S., Ting S.B., Watt M.J. (2019). The role of Ap2a2 in PPARα-mediated regulation of lipolysis in adipose tissue. FASEB J..

[86] Tsuchida A., Yamauchi T., Takekawa S., Hada Y., Ito Y., Maki T., Kadowaki T. (2005). Peroxisome proliferator–activated receptor (PPAR) α activation increases adiponectin receptors and reduces obesity-related inflammation in adipose tissue: Comparison of activation of PPARα, PPARγ, and their combination. Diabetes.

[87] Wang W., Lin Q., Lin R., Zhang J., Ren F., Zhang J., Ji M., Li Y. (2013). PPARα agonist fenofibrate attenuates TNF-α-induced CD40 expression in 3T3-L1 adipocytes via the SIRT1-dependent signaling pathway. Exp. Cell Res..

[88] Liu Z., Gan L., Wu T., Feng F., Luo D., Gu H., Liu S., Sun C. (2016). Adiponectin reduces ER stress-induced apoptosis through PPAR α transcriptional regulation of ATF2 in mouse adipose. Cell Death Dis..

[89] Tong Y., Hara A., Komatsu M., Tanaka N., Kamijo Y., Gonzalez F.J., Aoyama T. (2005). Suppression of expression of muscle-associated proteins by PPARα in brown adipose tissue. Biochem. Biophys. Res. Commun..

[90] Montagner A., Polizzi A., Fouché E., Ducheix S., Lippi Y., Lasserre F., Barquissau V., Regnier M., Lukowicz C., Benhamed F. (2016). Liver PPARα is crucial for whole-body fatty acid homeostasis and is protective against NAFLD. Gut.

[91] Hondares E., Rosell M., Diaz-Delfin J., Olmos Y., Monsalve M., Iglesias R., Villarroya F., Giralt M. (2011). Peroxisome proliferator-activated receptor α (PPARα) induces PPARγ coactivator 1α (PGC-1α) gene expression and contributes to thermogenic activation of brown fat: Involvement of PRDM16. J. Biol. Chem..

[92] Komatsu M., Tong Y., Li Y., Nakajima T., Li G., Hu R., Sugiyama E., Kamijo Y., Tanaka N., Hara A. (2010). Multiple roles of PPARα in brown adipose tissue under constitutive and cold conditions. Genes Cells.

[93] Miranda C.S., Silva-Veiga F., Martins F.F., Rachid T.L., Mandarim-De-Lacerda C.A., Souza-Mello V. (2020). PPAR-α activation counters brown adipose tissue whitening: A comparative study between high-fat–and high-fructose–fed mice. Nutrition.

[94] Barquissau V., Beuzelin D., Pisani D., Beranger G., Mairal A., Montagner A., Roussel B., Tavernier G., Marques M.-A., Moro C. (2016). White-to-brite conversion in human adipocytes promotes metabolic reprogramming towards fatty acid anabolic and catabolic pathways. Mol. Metab..

[95] Shen Y., Su Y., Silva F.J., Weller A.H., Sostre-Colon J., Titchenell P.M., Steger D.J., Seale P., Soccio R.E. (2020). Shared PPARα/γ target genes regulate brown adipocyte thermogenic function. Cell Rep..

[96] Rachid T.L., Penna-de-Carvalho A., Bringhenti I., Aguila M.B., Mandarim-de-Lacerda C.A., Souza-Mello V. (2015). Fenofibrate (PPARalpha agonist) induces beige cell formation in subcutaneous white adipose tissue from diet-induced male obese mice. Mol. Cell. Endocrinol..

[97] Defour M., Dijk W., Ruppert P., Nascimento E., Schrauwen P., Kersten S. (2018). The Peroxisome Proliferator-Activated Receptor α is dispensable for cold-induced adipose tissue browning in mice. Mol. Metab..

[98] Vosper H., Khoudoli G.A., Na Palmer C. (2003). The peroxisome proliferator activated receptor δ is required for the differentiation of THP-1 monocytic cells by phorbol ester. Nucl. Recept..

[99] Bastie C., Holst D., Gaillard D., Jehl-Pietri C., Grimaldi P.A. (1999). Expression of Peroxisome Proliferator-activated Receptor PPARδ Promotes Induction of PPARγ and Adipocyte Differentiation in 3T3C2 Fibroblasts. J. Biol. Chem..

[100] Hansen J., Zhang H., Rasmussen T.H., Petersen R.K., Flindt E., Kristiansen K. (2001). Peroxisome Proliferator-activated Receptor δ (PPARδ)-mediated Regulation of Preadipocyte Proliferation and Gene Expression Is Dependent on cAMP Signaling. J. Biol. Chem..

[101] Peters J.M., Lee S.S.T., Li W., Ward J.M., Gavrilova O., Everett C., Reitman M., Hudson L.D., Gonzalez F.J. (2000). Growth, Adipose, Brain, and Skin Alterations Resulting from Targeted Disruption of the Mouse Peroxisome Proliferator-Activated Receptor β(δ). Mol. Cell. Biol..

[102] Wang Y.-X., Lee C.-H., Tiep S., Yu R.T., Ham J., Kang H., Evans R. (2003). Peroxisome-Proliferator-Activated Receptor δ Activates Fat Metabolism to Prevent Obesity. Cell.

[103] Sodhi K., Puri N., Hyun K.D., Hinds T.D., Stechschulte L.A., Favero G., Rodella L., Shapiro J.I., Jude D., Abraham N.G. (2014). PPAR-delta binding to heme oxygenase 1 promoter prevents angiotensin II induced adipocyte dysfunction in goldblatt hypertensive rats. Int. J. Obes..

[104] Serrano-Marco L., Rodriguez-Calvo R., El Kochairi I., Palomer X., Michalik L., Wahli W., Vazquez-Cerrera M. (2011). Activation of Peroxisome Proliferator-Activated Receptor-β/-δ (PPAR-β/-δ) Ameliorates Insulin Signaling and Reduces SOCS3 Levels by Inhibiting STAT3 in Interleukin-6-Stimulated Adipocytes. Diabetes.

[105] Kang K., Reilly S., Karabacak V., Gangl M.R., Fitzgerald K., Hatano B., Lee C.-H. (2008). Adipocyte-Derived Th2 Cytokines and Myeloid PPARδ Regulate Macrophage Polarization and Insulin Sensitivity. Cell Metab..

[106] Pan D., Fujimoto M., Lopes A., Wang Y.-X. (2009). Twist-1 Is a PPARδ-Inducible, Negative-Feedback Regulator of PGC-1α in Brown Fat Metabolism. Cell.

[107] Lima R.T., Silva-Veiga F.M., Graus-Nunes F., Bringhenti I., Mandarim-de-Lacerda C.A., Souza-Mello V. (2018). Differential actions of PPAR-α and PPAR-β/δ on beige adipocyte formation: A study in the subcutaneous white adipose tissue of obese male mice. PLoS ONE.

[108] Mazuecos L., Pintado C., Rubio B., Guisantes-Batán E., Andrés A., Gallardo N. (2021). Leptin, Acting at Central Level, Increases FGF21 Expression in White Adipose Tissue via PPARβ/δ. Int. J. Mol. Sci..

